# Aseptic loosening after total hip arthroplasty and the risk of cardiovascular disease: A nested case-control study

**DOI:** 10.1371/journal.pone.0204391

**Published:** 2018-11-14

**Authors:** Agata Rysinska, Olof Sköldenberg, Anne Garland, Ola Rolfson, Sara Aspberg, Thomas Eisler, Göran Garellick, Andreas Stark, Nils Hailer, Max Gordon

**Affiliations:** 1 Karolinska Institutet, Department of Clinical Sciences at Danderyd Hospital, Stockholm, Stockholm, Sweden; 2 Department of Orthopaedics, Institute of Surgical Sciences, Uppsala University Hospital, Uppsala, Sweden; 3 Swedish Hip Arthroplasty Register, Gothenburg, Sweden; 4 Department of Orthopaedics, Institute of Clinical Sciences, Sahlgrenska Academy, Gothenburg, University of Gothenburg, Gothenburg, Sweden; 5 Harris Orthopaedic Laboratory, Massachusetts General Hospital, Boston MA, United States of America, and Harvard Medical School, Boston, MA, United States of America; Providence VA Medical Center, UNITED STATES

## Abstract

**Background:**

Patients with surgically treated osteoarthritis of the hip have an increased risk of cardiovascular morbidity and mortality many years after the operation compared with controls. Our hypothesis is that this increased risk after total hip arthroplasty (THA) is mediated by development of periprosthetic osteolysis leading to aseptic loosening of the implant.

**Methods:**

We conducted a nation-wide, nested, case-control study consisting of patients receiving a cemented THA due to osteoarthritis between the years 1992 and 2005. Our study population included a total of 14,430 subjects identified in the Swedish hip arthroplasty register and linked to the Swedish National Patient Register. The case group consisted of patients (n = 2,886) who underwent reoperation of the treated hip due to osteolysis or aseptic loosening at any time within five years after the index surgery. Each case was matched with four controls (n = 11,544) who had not undergone reoperation. The main outcomes were cardiovascular events i.e. myocardial infarction, heart failure and cerebral infarction according to ICD-codes and time to the first cardiovascular event during the exposure period. Outcomes were subgrouped into cardiac and cerebral events. We used regression models to calculate the incidence rates and adjusted our results for confounders.

**Findings:**

Overall, 5.1% of patients had cardiac events, with slightly more overall cardiovascular events occurring in the control group (8.1% vs. 6.7%, odds ratio 0.8, 95% confidence interval (CI) 0.7 to 1.0). After adjusting for confounders, the case group had an increased relative risk of 1.3 (95% confidence interval (CI) 1.1 to 1.3) for total number of cardiovascular events. Similar effect sizes were observed for time to first event.

**Interpretation:**

Patients with osteoarthritis who received THA and subsequently underwent a revision operation due to loosening had a higher relative risk of developing cardiovascular events than controls. Thus there is an association which could be explained by a common inflammatory disease pathway that requires further experimental research.

## Putting research into context

### Evidence before the study

Total hip arthroplasty (THA) is a common and important treatment for osteoarthritis (OA) patients. Long-term cardiovascular effects elicited by OA or the implant itself remain unknown. In a previous study, we have shown that there is an increased risk of late cardiovascular morbidity and mortality after THA.

### Added value of this study

Our hypothesis is that this increased risk many years after total hip arthroplasty (THA) is mediated by development of periprosthetic osteolysis leading to aseptic loosening of the implant. Patients with osteoarthritis who received THA and subsequently underwent a revised operation due to loosening and/or osteolysis had a 30% higher relative risk of developing cardiovascular events than controls. This may be indicative of a common inflammatory disease pathway that requires further experimental research.

### Implications of all the available evidence

Patients with osteoarthritis who received THA and subsequently underwent revision surgery due to aseptic loosening/osteolysis had a higher relative risk of developing cardiovascular events than controls. This may be suggestive of a common inflammatory disease pathway that requires further experimental research.

## Background

Although it is a remarkably successful surgical procedure, patients treated with total hip arthroplasty (THA) for osteoarthritis have an increased risk of cardiovascular morbidity and mortality compared to healthy controls one decade after surgery.[1, 2] This observation may be indicative of an inflammatory response due to the prosthetic implant. As the implant ages, debris is generated in the artificial joint, triggering an inflammatory foreign-body tissue reaction and peri-implant bone resorption known as osteolysis and aseptic loosening.[3, 4] Particles derived from the polyethylene liner, metal ions, and metallic nanoparticles have all been associated with the development of aseptic loosening and soft-tissue reactions. Moreover, aseptic loosening of the implant is the most common reason for revision surgery.[5]

The link between osteolysis and cardiovascular disease may be mediated by the signalling pathway receptor activator of nuclear factor κ B (RANK)/RANK-ligand (RANKL)/OPG. This pathway is activated during vascular calcification because a disturbance in RANK/OPG can increase calcification in blood vessels.[6–8] Furthermore, increasing evidence suggests that both osteopenia and vascular calcification may be linked.[7] Clinical associations between coronary artery disease and serum RANKL levels have also been described.[9, 10] Therefore, RANKL/OPG are recognized as having significance for arterial calcification as well as osteolysis in bone.[7] In addition to associations of cardiovascular diseases with bone and joint conditions, it is possible that the orthopaedic implant in itself can cause local and systemic inflammation that is generally believed to increase the risk of cardiovascular disease.[11–13]

In a previous study, we found an increased long-term risk of cardiovascular mortality in patients treated with THA.[2] In the current study, we hypothesize that this late cardiovascular risk following THA may be mediated by the development of osteolysis and systemic inflammation.

## Methods

### Design and setting

The study design is a nation-wide, nested, case-control study. Ethical approval was obtained by the Karolinska Institute. During the study period of 1992 to 2005, the yearly average Swedish population was 8.9 million residents, and the study base consisted of patients receiving a cemented THA due to primary hip osteoarthritis during this time period.[14] We only included patients that had received cemented total hips with metal-on polyethylene bearings. In Sweden, highly-crosslinked polyethylene was introduced in 2005 and thus the population consists primarily of standard polyethylene. During the period both modular and non-modular necks were used. Follow-up data on deaths, causes of death, admissions to inpatient care, reasons for inpatient care admission and reoperations were collected until 2012.[15] Only the first hip was included for patients who underwent bilateral surgery.

### Participants

#### Cases

Cases were defined as any patients who had undergone reoperation of the treated hip due to aseptic loosening at any time point.

**Fig 1:**
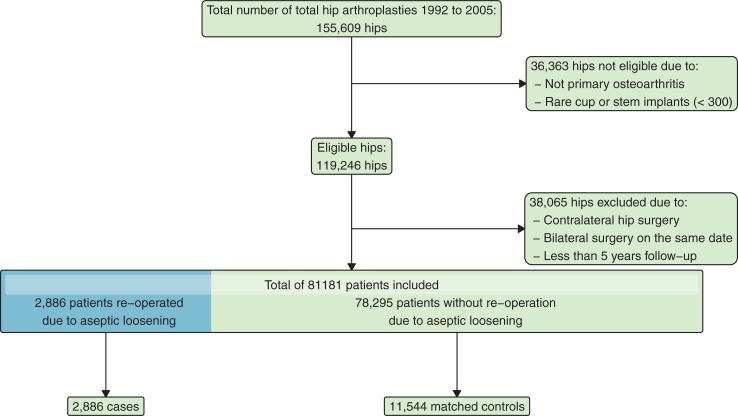
Directed acyclic graph (DAG) for identifying confounders and minimizing bias prior to the start of the study.

**Controls:** The cases were then matched 4 to 1 with unique patients from the Swedish Hip Arthroplasty Register (SHAR). We matched controls that had the same exposure time for cardiovascular events e.g. we would match a case with 8 years before aseptic loosening randomly to a control that had at least survived 8 years since their surgery. After matching, we assigned to the controls the same exposure time as their corresponding case. We only included cardiovascular events during that exposure period. All units (1 case, 4 controls) with an exposure time of less than 6 years were excluded from the study (Fig 1).

### Data sources

The SHAR was founded in 1979 and provides prospective, observational, nationwide data on hip arthroplasties. Since 1992, personal identity numbers have been collected, allowing for patient-specific follow-up with a capture rate of 97%. The registry is the second oldest arthroplasty quality register in the world and includes 98% of all patients who undergo hip arthroplasty in all Swedish hospitals.[16]

To calculate the comorbidity measures and outcomes, the patients were cross-matched, using the personal registration number, with data from the Swedish National Patient Register (NPR). The NPR was started in 1964 and includes all inpatient care in Sweden since 1987 with ICD-9 and ICD-10 discharge codes together with admission and discharge dates. The proportion of patients with a given diagnosis where the registry code is deemed correct (positive predictive value) is estimated to be 85%-90%.[17]

### Exposure

The exposure period was defined as the period between 5 years after the first surgery and 1 year before the first re-operation due to aseptic loosening. The initial 5 years were regarded as an incubation period in which the process of aseptic loosening may have been initiated but was unlikely to influence cardiovascular morbidity. We excluded the last year before surgery because patients with symptomatic loosening of the implant due to aseptic loosening may risk poor ambulatory function leading to increased cardiovascular risk.

### Outcome measures

The main outcomes of interest were the following cardiovascular events: myocardial infarction, heart failure and cerebral infarctions according to ICD codes (Table 1) and time to first cardiovascular event during the exposure period. Only admissions of a minimum of 2 days in duration were included, and only the first admission was analysed in the case of multiple admissions to avoid the same event appearing multiple times. Outcomes were subgrouped into cardiac and cerebral categories.

**Table 1:** Outcome codes. Note that ICD 9 and 10 differ, and some groups lack a corresponding category for ICD 9/10.

Group	ICD 9	ICD 10
Cardiac		
Unstable angina	411.1, 411.9	I20.0
Acute infarction	-	I21.0–4, I21.9
Re-infarction	-	I22.0, I22.2, I22.8–9
Atherosclerotic disease	414.0, 414.8, 414.9	I25.0–1
Quiet ischaemia	-	I256
Old infarction	412	-
Cerebral		
Thrombotic or embolic	-	I63.0–2, I63.4–5, I63.8–9
Occlusion, pre-cerebral	433.0–3	-
Occlusion, cerebral	434.0, 434.9	-

### Confounders

We used a directed acyclic graph (see S1 Fig) to determine the confounders. We adjusted for cardiovascular comorbidity by the number of cardiovascular events that occurred between 0.5 and 5 years after surgery and for chronic heart failure according to the Charlson definition.[18] The cardiovascular event count was outcome-specific, i.e., when adjusting for cardiac events, only cardiac events during the first 5 years were counted, and when adjusting for cerebral events, only cerebral events were used. We further adjusted for chronic obstructive lung disease (COPD) as a proxy for smoking, Charlson comorbidity index,[18] age as a general confounding comorbidity[19] and calendar year; the latter was used primarily due to a change in outcome estimates (i.e., changed practices of ICD coding) during the study period. We also adjusted for sex because we deemed it probable that this may affect mobility, inflammatory responses, and outcomes.

### Statistical methods

We used Poisson regression for the time to event data with a log offset term for the exposure time until the first event to estimate the rate. The Poisson regression assumes that the variance and the mean count are equal. We found that the time to event did not violate the variance assumption, while the number of events did violate this assumption. We therefore used a negative binomial regression model to calculate the relative risks for the counts. The negative binomial is a generalization of the Poisson regression with an additional frailty parameter. We used the same offset model as described for the Poisson regression.

All continuous variables, age and calendar year, were tested for non-linearity. If non-linearity was indicated by a likelihood ratio test resulting in a p-value below 0.05, the variable was modelled with a restricted cubic spline. To avoid overfitting the regression model by choosing too many knots, the number of spline knots was chosen using the Bayesian information criterion.

All analyses were performed using R 3.4.3, the MASS package for negative binomial regression (v 7.3–48), the AER package for investigating the variance assumption (overdispersion) for Poisson regression (v 1.2–5), knitr (v. 1.19) for reproducible research, Gmisc (v. 1.5) with Greg (v. 1.2) for table output.

## Results

The study sample consisted of 2,886 cases and 11,544 matched controls (Fig 1). The mean follow-up time was 9.8 years, and the longest follow-up was 19.8 years. At 5, 7.5 and 10 years after surgery, 88.3%, 79.5% and 68.9% were still alive. The differences between the case and control groups were statistically significant at baseline; the control group had more females, was an average of 5 years older than the case group, and had a considerably higher comorbidity burden (Table 2).

**Table 2:** Study population characteristics. Continuous variables are presented as the mean and standard deviation. The control group generally exhibited a higher co-morbidity prevalence, and the outcomes were similarly distributed.

Variable	Control group No. 11,544	Osteolysis group No. 2,886	P-value
Age	68.4 (±8.3)	63.1 (±8.0)	< 0.0001
Female	7,133 (61.8%)	1,396 (48.4%)	< 0.0001
Calendar year	1997.4 (±3.5)	1996.8 (±3.4)	< 0.0001
Comorbidity†			0.081
Myocardial infarction	160 (1.4%)	28 (1.0%)	
Heart failure	93 (0.8%)	12 (0.4%)	0.027
Hypertension	29 (0.3%)	5 (0.2%)	0.53
Diabetes	23 (0.2%)	8 (0.3%)	0.38
Peripheral vascular disease	70 (0.6%)	6 (0.2%)	0.006
Cerebrovascular disease	240 (2.1%)	36 (1.2%)	0.003
Chronic pulmonary disease	55 (0.5%)	5 (0.2%)	0.022
Rheumatic disease	49 (0.4%)	2 (0.1%)	0.002
Peptic ulcer	30 (0.3%)	8 (0.3%)	0.84
Malignancy	158 (1.4%)	29 (1.0%)	0.14
Charlson’s index			0.0004
None	11,166 (96.7%)	2,830 (98.1%)	
1–2	363 (3.1%)	54 (1.9%)	
≥ 3	15 (0.1%)	2 (0.1%)	
Mean (SD)	0.06 (±0.34)	0.04 (±0.27)	0.0002
Number of cardiovascular admissions (0.5–5 years)			0.0005
None	10,605 (91.9%)	2,693 (93.3%)	
1–2	848 (7.3%)	186 (6.4%)	
≥ 3	91 (0.8%)	7 (0.2%)	
Number of cardiac admissions (0.5–5 years)			0.068
None	10,950 (94.9%)	2,740 (94.9%)	
1–2	529 (4.6%)	139 (4.8%)	
≥ 3	65 (0.6%)	7 (0.2%)	
Number of cerebral admissions (0.5–5 years)			< 0.0001
None	11,157 (96.6%)	2,831 (98.1%)	
1–2	367 (3.2%)	55 (1.9%)	
≥ 3	20 (0.2%)	0 (0.0%)	

† A subset of comorbidities as defined by the Charlson and Elixhauser comorbidity indices

## Main results

**Fig 2:**
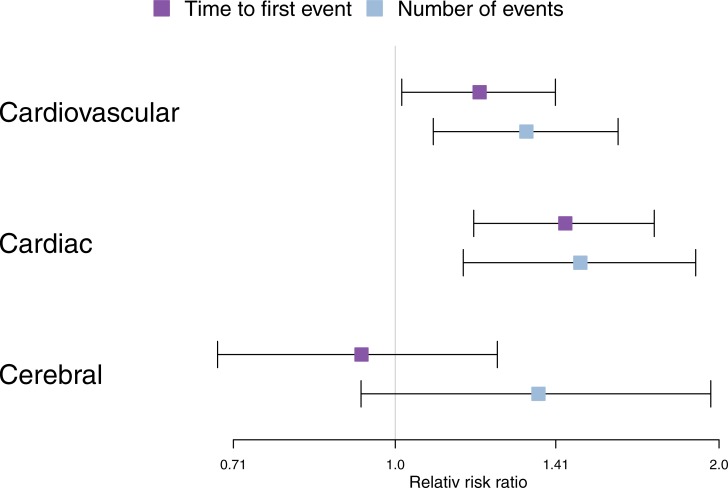
Forest plot comparing the different groups and outcomes.

Before adjusting for confounders, the control group experienced slightly more cardiovascular events, 8.1% vs 6.7% (odds ratio 0.8, 95% confidence interval (CI) 0.7 to 1.0), while in both groups, 5.1% of participants had cardiac events. The mean time to the first event, after the incubation period, was less than 10 years from surgery and did not differ between the groups (Table 3). When adjusting for confounders, we found that the case group had an increased relative risk of 1.3 (CI 1.1 to 1.3) for total number of cardiovascular events (Table 4). This effect was primarily mediated via cardiac events, with a risk of 1.5 (95% CI 1.2 to 1.7). Similar effect sizes were observed for time to first event (Fig 2). The relative risk of developing chronic heart failure for cases was increased compared with that in the control group (Table 3).

**Table 3:** Types and number of admissions. Continuous variables are presented as the mean and standard deviation. The control group generally exhibited a higher co-morbidity prevalence, and the outcomes were similarly distributed. The mean time is after excluding the first 5 years.

Admission type	Control group No. 11,544	Osteolysis group No. 2,886	P-value
Cardiovascular admissions (≥ 5 years)			
Event	939 (8.1%)	193 (6.7%)	
Mean time to event	9.6 (±3.1)	9.6 (±3.1)	0.81
Cardiac admissions (≥ 5 years)			
Event	594 (5.1%)	146 (5.1%)	
Mean time to event	9.7 (±3.1)	9.6 (±3.1)	0.76
Cerebral admissions (≥ 5 years)			
Event	387 (3.4%)	55 (1.9%)	
Mean time to event	9.7 (±3.1)	9.7 (±3.1)	0.60

**Table 4:** The fully adjusted relative risk ratios. Both models are adjusted by all variables within the table with additional adjustments for calendar year and age. Age and calendar year are not displayed due to non-linearity.

	Overall		Cardiac		Cerebral	
Variable	RR	95% CI	RR	95% CI	RR	95% CI
**First event†**						
Group						
Control	1.0	ref	1.0	ref	1.0	ref
Osteolysis	1.2	1.0 to 1.4	1.4	1.2 to 1.7	0.9	0.7 to 1.2
Sex						
Female	1.0	ref	1.0	ref	1.0	ref
Male	1.2	1.0 to 1.3	1.3	1.1 to 1.5	0.9	0.8 to 1.2
Chronic pulmonary disease						
No	1.0	ref	1.0	ref	1.0	ref
Yes	2.2	1.0 to 4.2	2.1	0.8 to 4.6	1.9	0.5 to 5.5
Heart failure						
No	1.0	ref	1.0	ref	1.0	ref
Yes	1.2	0.5 to 2.8	1.6	0.6 to 4.1	0.7	0.1 to 2.9
Charlson index	1.1	0.8 to 1.4	1.1	0.8 to 1.5	1.2	0.8 to 1.6
No. adm. 0.5–5 years	2.1	2.0 to 2.1	2.4	2.3 to 2.5	2.8	2.7 to 2.9
**Total No. of events‡**						
Group						
Control	1.0	ref	1.0	ref	1.0	ref
Osteolysis	1.3	1.1 to 1.6	1.5	1.2 to 1.9	1.4	0.9 to 2.0
Sex						
Female	1.0	ref	1.0	ref	1.0	ref
Male	1.2	1.1 to 1.4	1.4	1.2 to 1.7	1.0	0.8 to 1.3
Chronic pulmonary disease						
No	1.0	ref	1.0	ref	1.0	ref
Yes	1.7	0.6 to 4.1	1.5	0.4 to 4.8	1.2	0.2 to 6.0
Heart failure						
No	1.0	ref	1.0	ref	1.0	ref
Yes	1.3	0.5 to 3.7	1.4	0.4 to 4.6	1.3	0.1 to 7.8
Charlson index	1.0	0.7 to 1.4	1.2	0.8 to 1.6	1.1	0.6 to 1.8
No. adm. 0.5–5 years	9.3	8.5 to 10.3	13.9	12.2 to 15.9	49.9	39.2 to 64.4

† Poisson regression was used to calculate the incidence rate based on the time to first occurrence.

‡ Negative binomial regression was used to calculate the number of incidences until 1 year prior to the revision for osteolysis for the case group and the corresponding time period for the control group.

## Discussion

### Main findings

In this nation-wide, nested, case-control study on THA patients, we show that patients who develop symptomatic osteolysis and loosening of the implant have a higher risk of cardiovascular morbidity than controls and that the risk increase is primarily driven by cardiac events. The control group had a higher burden of comorbidities, but after adjusting for possible confounders, we found an approximately 50% increase in the relative risk of cardiac events among the case group. The effect was not seen for the cerebral event, but due to the wide confidence interval, this result should be interpreted with caution. The process leading to osteolysis/loosening in most cases starts much earlier than one year before a revision operation. Symptoms may be present for many years, which in turn may influence the patient’s physical activity level and risk for cardiovascular events. Fig 2.

THA is the most successful and efficient procedure for treating osteoarthritis of the hip. Twenty years after index surgery, approximately 90% of implants are still well-functioning.[5] The most common reason for revision surgery is aseptic loosening, which is very often accompanied by osteolysis.[1] Wear particles generated from the polyethylene bearings of hip prostheses have long-been recognized as causative of periprosthetic osteolysis.[20] Such periprosthetic osteolysis can induce loosening of the implant, but wear-associated osteolysis is a rather slow process that rarely occurs before five years after the index surgery.[21] Osteolysis has also been attributed to fluid movement and pressure at the prosthesis-bone interface, which can induce expression of pro-inflammatory mediators that act as activators of osteoclasts.[22]

The inflammatory process that drives osteolysis is in part mediated by the RANK/OPG signalling pathway, which plays a crucial role in regulating osteoclast function.[23] In a retrospective cohort study of patients who underwent THA, the sum of osteolytic areas defined on plain radiography was correlated with concentrations of RANKL in patient blood, indicative of the systemically detectable involvement of this pathway in the process of periprosthetic osteolysis.[24]

The above described signalling pathway is also involved in development of atherosclerosis. The calcification of the media layer in arterial vessels is mediated by RANK/OPG, and thus, the RANK/OPG system may represent a common pathway that plays a role in both osteolysis and atherosclerosis.[25] This putative molecular link between calcifying atherosclerosis and osteolysis could explain the association between these two disease entities found in our present study. It remains to be determined whether periprosthetic osteolysis triggers inflammatory processes in vessels and thus contributes to the development of atherosclerosis or whether patients suffering from both osteolysis and atherosclerosis have an underlying propensity to develop low-key inflammation at several sites. In addition, further common inflammatory pathways could be involved in the development of both diseases.

Arthrosis and cardiovascular disease have common risk factors. In this study, we discuss inflammation but there are other plausible mechanisms. Age and high BMI increases the risk for both osteoarthrosis and cardiovascular disease. Decreased physical activity increases the risk for high BMI, diabetes and cardiovascular disease. Reversed relationship is also possible, where cardiovascular disease could play a role in the development and the progression of arthrosis. One possible mechanism could be reduced capillary flow in the subchondral bone which leads to defective supply of nutrition to the cartilage, which in turn leads to inferior quality and loss of the same.

Another plausible explanation could be the pain treatment of osteoarthritis. Oral NSAID gives pain relief, but unfortunately also raises the risk for myocardial infarction and stroke. The correlations between osteoarthrosis and cardiovascular disease are complex and likely there are several explanations.

### Limitations and strengths of the study

Our study has several limitations. Only patients who proceeded to revision surgery were included in the case group. All revisions are directly reported to the Swedish hip arthroplasty registry. The reports consist of medical records including (1) the surgical and (2) the discharge record. These are manually classified by a small set of trained secretaries at the Swedish Hip arthroplasty registry into specific causes. There are no radiographs available for the classification allowing some misclassification bias but this should be negligible.

We were unable to identify patients with aseptic loosening who were unfit for revision surgery, which may result in a potential misclassification bias and/or a so called immortal time bias. The effect of these limitations, if present in significant numbers, would decrease rather than increase the effect size as more actual cases (with aseptic loosening) who were too sick to undergo surgery would be incorrectly classified as controls. Furthermore, registry data lack the internal validity of well-defined prospective cohorts. However, both registries used in the current study have good accuracy and completeness, and the positive predictive value of the NPR has been estimated at 90±5%, indicating high validity of the data.[17] Swedish personal registration numbers ensure that patients can be followed throughout the study period until an event, death, or emigration. In the diagnosis of cardiovascular disease, a potential surveillance bias exists that can lead to detection bias. Patients with symptomatic aseptic loosening may experience pain from a previously well-functioning THA procedure, and thus be more likely to contact healthcare providers and be diagnosed with other conditions, such as cardiovascular disease. This would increase the effect estimate for the case group. However, the definition of our primary outcome was strict, with a requirement of admission to a hospital for at least 2 days due to cardiovascular events for classification as a cardiovascular event. This definition excludes en-passant registration of cardiovascular diagnoses and thereby reduces detection bias. In addition, we attempted to minimize detection bias by only including the time period up to one year prior to revision surgery, thus eliminating pre-operative diagnosis of cardiovascular disease in cases subject to osteolysis/loosening.

This study also lacked adjustment for obesity and smoking, as these data were unavailable in both the SHAR and the NPR during the studied time period. Obesity increases the risk of cardiovascular disease but does not appear to affect the risk of developing osteolysis.[26] However, the question of under-diagnosed obesity remains a limitation of our study. Smoking increases the risk of cardiovascular disease, but no data on smoking habits were available. We therefore used COPD as a proxy to detect smoking habits in our cohort but found no support for a higher prevalence of smoking within the loosening/osteolysis group.

Our study also has several strengths. The Swedish nationwide, health care registers are of high quality and offer excellent opportunities for epidemiological research.[17] Our cohort of THA patients has the longest follow-up period ever published based on a population-wide sample. To our knowledge, the analysis of the risk of cardiovascular admission to inpatient care among hip arthroplasty patients has only rarely been investigated previously.[2, 27] Recently, Wyles and co-authors have reported results from a autopsy-study on patients with and without joint replacement and found increased cardiac cobalt levels in concert with cardiomegaly and increased interstitial fibrosis. In addition, these cobalt levels were 69% higher in patients who had undergone THA revision than in those who underwent primary THA.[28] This further supports the results from our large cohort where patients with later revision of their artificial joint suffer more cardiac events and the mechanism for this finding can thus have several explanations. In addition, the findings presented in the current study are consistent with the previously reported incidence of admissions to hospital care due to cardiovascular reasons in THA patients and matched control individuals.

### Conclusion

In conclusion, patients who receive a THA and subsequently undergo revision surgery due to loosening and/or osteolysis have a 30% higher relative risk of developing cardiovascular events than controls. This may be indicative of a common inflammatory disease pathway that requires further experimental elucidation.

## Supporting information

S1 Fig Directed acyclic graph (DAG) for identifying confounders and minimizing bias prior to the start of the study. (EPS)
